# Crossover versus Stabilometric Platform for the Treatment of Balance Dysfunction in Parkinson's Disease: A Randomized Study

**DOI:** 10.1155/2015/878472

**Published:** 2015-10-25

**Authors:** G. Frazzitta, F. Bossio, R. Maestri, G. Palamara, R. Bera, D. Ferrazzoli

**Affiliations:** ^1^Department of Parkinson Disease and Brain Injury Rehabilitation, “Moriggia-Pelascini” Hospital, Gravedona ed Uniti, 22015 Como, Italy; ^2^Department of Biomedical Engineering, Scientific Institute of Montescano, S. Maugeri Foundation IRCCS, Montescano, 27040 Pavia, Italy

## Abstract

Balance dysfunctions are a major challenge in the treatment of Parkinson's disease (PD). Previous studies have shown that rehabilitation can play a role in their treatment. In this study, we have compared the efficacy of two different devices for balance training: stabilometric platform and crossover. We have enrolled 60 PD patients randomly assigned to two groups. The first one (stabilometric group) performed a 4-week cycle of balance training, using the stabilometric platform, whereas the second one (crossover group) performed a 4-week cycle of balance training, using the crossover. The outcome measures used were Unified Parkinson's Disease Rating Scale (UPDRS) part II, Berg Balance Scale (BBS), Timed Up and Go (TUG), and Six Minutes Walking Test (6MWT). Results showed that TUG, BBS, and UPDRS II improved in both groups. There was not difference in the efficacy of the two balance treatments. Patients in both groups improved also the meters walked in the 6MWT at the end of rehabilitation, but the improvement was better for patients performing crossover training. Our results show that the crossover and the stabilometric platform have the same effect on balance dysfunction of Parkinsonian patients, while crossover gets better results on the walking capacity.

## 1. Introduction

Balance dysfunction (BD) in Parkinson's disease (PD) is a disabling symptom that contributes to falls and it impairs the ability to perform the activities of daily living, such as walking, turning, and rising to a standing position [1].

Even with optimal pharmacological and surgical management, these deficits cannot be controlled satisfactorily and have a negative impact on the quality of life [2]. There is an increasing evidence that physical therapy, especially highly challenging balance exercises, can improve BD and reduce the risk of falls, though the long-term effects of physical therapy interventions need to be further explored [3, 4]. Previous studies have shown the efficacy of training with stabilometric platform and visual feedback for balance in Parkinsonian patients in stage 3 of Hoehn-Yahr scale [5]. Unfortunately, the stabilometric platforms are not widespread and they are hard to find in the normal gymnasiums, while the crossover is widely used. Crossover is a device that simulates the movement of a skater, broadening the support base and improving the functionality of paravertebral muscles. Therefore, this device can act on several fundamental aspects of PD such as the narrow-based gait [6], the loss of automatisms (e.g., upper arms swinging) [7], and the altered functionality of the trunk muscles [8]. With its automatic and coordinate movement of upper and lower limbs, crossover could help patients in the unconscious relearning of upper limb swinging and it could also improve the narrow base support [9]. Furthermore, the wide dissemination of crossover could allow Parkinsonian patients an early, continuous, and cheap treatment that could have beneficial effects both on the development and on the control of BD.

The aim of this study was to assess the efficacy of crossover compared to the stabilometric platform in the treatment of BD in patients with PD in order to propose the crossover as a useful device for their treatment.

## 2. Methods

From January to October 2014, we screened one hundred Parkinsonian patients referred to the Department of Parkinson's disease Rehabilitation of the “Moriggia-Pelascini” Hospital (Gravedona ed Uniti, Italy).

Inclusion criteria were (1) probable diagnosis of PD according to Gelb et al. [10], (2) stage 3 of Hoehn-Yahr scale, (3) ability to walk without any assistance, and (4) mini mental state examination > 25.

Exclusion criteria were (1) presence of dyskinesias, (2) presence of other neurological diseases, (3) visual and/or auditory dysfunctions that impair gait and balance, (4) postural hypotension, and (5) severe orthopaedic and/or rheumatic diseases.

Patients were randomly assigned to two groups: group 1 (stabilometric group) underwent a 4-week cycle of stabilometric platform training for 6 days per week and group 2 (crossover group) underwent a 4-week cycle of crossover training for 6 days per week.

For the patients' randomization, a computer-generated list of binary random numbers was used. Both groups were evaluated at admission and at discharge, using the following outcome measures: Berg Balance Scale (BBS), Timed Up and Go (TUG) Test, Six Minutes Walking Test (6MWT), and Unified Parkinson's Disease Rating Scale part two (UPDRS II), daily activities.

Patients were evaluated at 9 A.M., one hour after taking the dopaminergic replacement therapy (including levodopa, dopamine agonists, and monoamine oxidase-B inhibitors). A neurologist, expert in movement disorders, and a physiotherapist, both blind to the purpose of the study, evaluated all patients. The study design and protocol were approved by the local Scientific Committee and Institutional Review Board (General Hospital Moriggia Pelascini, Gravedona e Uniti-Como) and followed the ethical principles outlined by the Helsinki Declaration.

### 2.1. Sample Size Computation

Published studies report a standard error of measurement (SEM) equal to 1.8, 0.59 s, and 30 m for BBS, TUG, and 6MWT, respectively [11, 12]. We wanted to detect a difference in improvement between groups of 2, 1 s, and 35 m, respectively. To detect these differences with a two-tailed type I error of 0.05 and a power of 80%, the estimated sample size was of 27, 12, and 24 patients per group, respectively. Taking into account the possibility of a small rate of dropout, we set our sample size to 30 + 30 patients.

### 2.2. Outcome Measures

The efficacy of the two different treatments was evaluated with the following outcome measures.

#### 2.2.1. Berg Balance Scale (BBS)

The Berg Balance Scale (BBS) is the gold standard scale for balance tests. It is a 14-item test designed to measure the balance during specific functional tasks. Each task is scored from 0 to 4 for a maximum of 56 points (normal subject).

#### 2.2.2. Six Minutes Walking Test (6MWT)

In the 6MWT, subjects walk as far as they can for 6 minutes. Patients are allowed to take a rest or slow down during the test (if they need it). We use for the test an unimpeded hallway length 15 meters in order to increase the number of turnings during walking, being the turning one of the major problems of Parkinsonian patients.

#### 2.2.3. Timed Up and Go Test

This test measures mobility in elderly people and it is a useful tool to quantify locomotion performance in individuals with PD. The patients have to stand up from a chair, walk 3 meters, turn around, walk back to the chair, and sit down again. The whole test is repeated 3 times and the average time is calculated.

#### 2.2.4. UPDRS II

The UPDRS is a common scale used to follow the longitudinal course of PD. Part II of this section assesses the activities of daily life including speech, swallowing, handwriting, dressing, hygiene, falling, salivating, turning in bed, walking, and cutting food.

### 2.3. Characteristics of Devices

#### 2.3.1. The Stabilometric Platform

**Figure 1:**
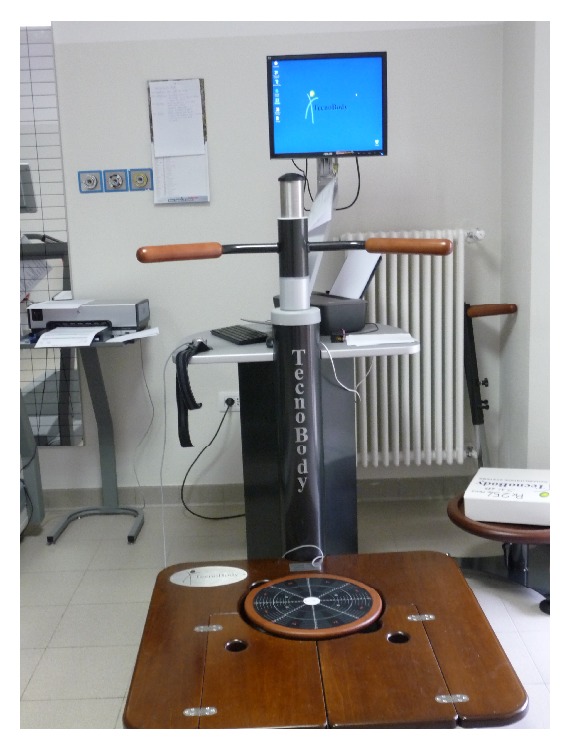
Stabilometric platform.

For the training of PD patients, we have used a stabilometric platform (Prokin 254 (Pro-Kin Software Stability), TecnoBody S.R.L., Dalmine, 24044 Bergamo, Italy). This device is a force platform with a flat and regular surface fixed to four force-transduction systems (Figure 1). The platform sends the signals to a computer for offline analysis and for detecting the position of the centre of pressure (CoP). The CoP represents the point of application of forces concerning feet and ground. The CoP area is an index of the effectiveness of the tonic postural system in keeping the centre of gravity closer to the intermediate position of balance.

#### 2.3.2. Crossover

**Figure 2:**
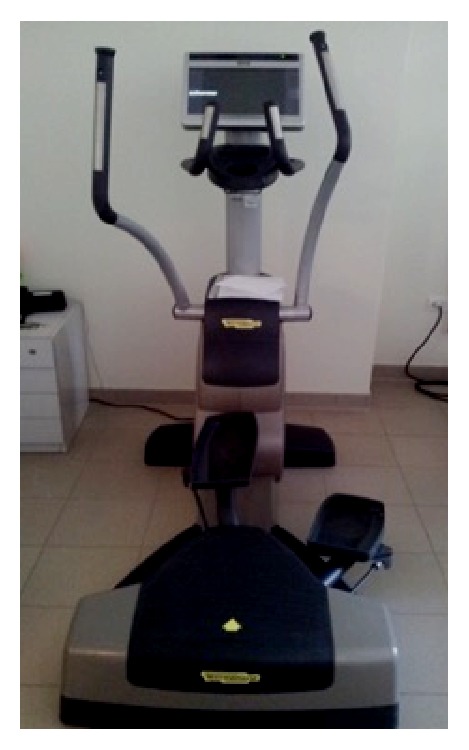
Crossover.

Crossover is a type of cross-trainer, designed for cardiovascular exercises. It has two platforms and two interconnected levers that move simultaneously. It engages the arms in a converging motion and the legs in extending, rotating, and flexing actions so that multiple muscles are working at the same time (Figure 2).

This device does not have feedback. There are 25 levels of difficulty based mainly on the progressive increase of resistance of the footboards.

### 2.4. Rehabilitative Treatment

All patients underwent a front-to-front treatment of 20 minutes with a physiotherapist, performing cardiovascular exercises, stretching exercises, and a passive mobilization of four limbs. Then patients underwent a stabilometric platform training or a crossover training for 15 minutes.

#### 2.4.1. Stabilometric Group

**Figure 3:**
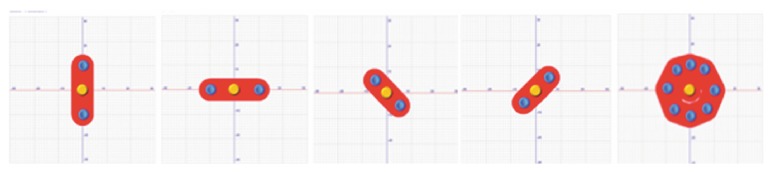
Examples of the five “stabilometric track” exercises.

**Figure 4:**
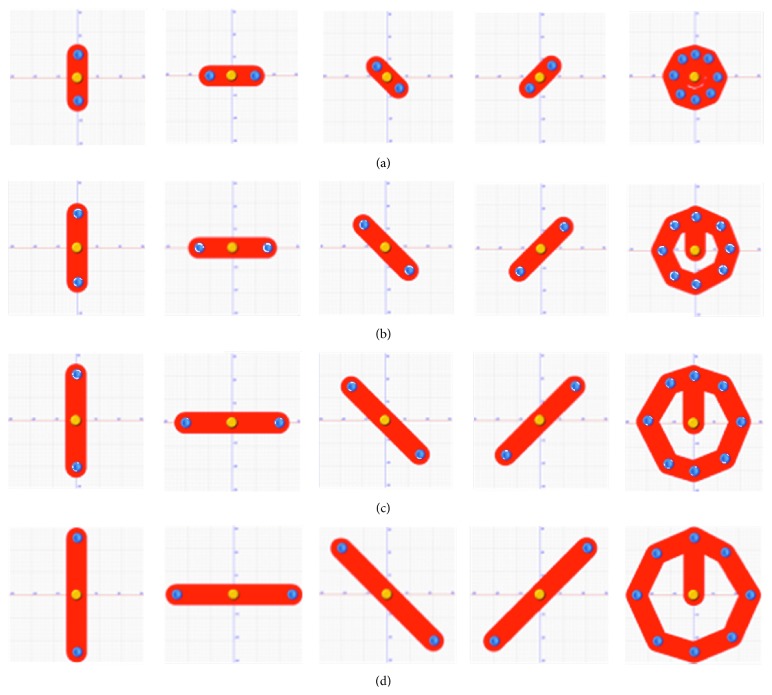
The exercises on the platform increased in difficulty each week and were studied in order to progressively stress the patient's limit of stability. (a) Exercises performed on the 1st week. (b) Exercises performed on the 2nd week. (c) Exercises performed on the 3rd week. (d) Exercises performed on the 4th week.

Subjects underwent a 4-week cycle of balance training using stabilometric platform (Prokin 254, TecnoBody S.R.L., Dalmine, 24044 Bergamo, Italy) for 6 days per week. The patients performed five “stabilometric track” exercises (see Figure 3). The patients had to stand still on a force plate with their feet comfortably positioned within a box whose dimensions are equal to their foot length. They looked straight ahead at a screen surface placed 80 cm away, putting their arms on two handles. Using a visual feedback sensitive to the displacement of the centre of gravity, patients had to move their CoP within a red track in order to reach two yellow circles placed at the end of the track. Patients were not supposed to leave the track and an auditory feedback signalled any possible deflection. The five tracks allowed different positions in the space (vertical, horizontal, oblique (left/right), and circular) (Figure 3). The duration of each exercise increased from 30′′ on the first week to 60′′ on the second week and to 90′′ on the third week until it reached 120′′ on the last week. Each exercise was performed only one time on the 1st week and on the 2nd week and 2 times on the 3rd week and on the 4th week. A pause between the 4th and 5th exercise was programmed on the first two weeks and between the 3rd and 4th exercise on the two final weeks. The exercises on the platform increased in difficulty each week and were studied in order to progressively stress the patient's limit of stability (i.e., every week the length of the tracks was increased until a designed target in different space directions—antero-posterior, medio-lateral direction, etc.—was chosen) (Figure 4).

#### 2.4.2. Crossover Group

Each patient underwent a 4-week cycle of crossover training (Technogym Crossover 700) for 6 days per week. In our study, the crossover training involved only the first 4 of the 25 levels of difficulty/resistance: first week, level 1; second week, level 2; third week, level 3; and fourth week, level 4. Patients performed a 5-minute treatment and repeated it 3 times with a 5-minute break between each repetition. The use of the first 4 levels on the crossover is due to the need to maintain the exercises on aerobic conditions.

### 2.5. Statistical Analysis

Descriptive statistics are reported as mean (SD). The normality of the distribution of all variables was assessed by the Shapiro-Wilk statistic. For each outcome variable considered, the effect of the two different rehabilitation protocols was assessed by a two-factor analysis of variance: the first factor was the rehabilitation protocol (stabilometric platform versus crossover) and the second factor was time (end of treatment versus baseline) with repeated measures of the time factor. If a significant interaction effect for time × treatment was found, two separate paired *t*-tests (one for each group of patients) were carried out to compare end of rehabilitation and baseline values.

Between-group comparisons for continuous data were assessed with unpaired *t*-test or with Mann-Whitney *U* test in case of violation of the normality assumption. Comparisons for categorical variables were carried out by the Chi-square test or Fisher's exact test when appropriate.

A *p* value < 0.05 was considered statistically significant. When multiple comparisons were carried out, the Bonferroni correction was applied. Accordingly, when couples of comparisons were considered, the significance level was set to 0.025. All analyses were carried out using the SAS/STAT statistical package, release 9.2 (SAS Institute Inc., Cary, NC, USA).

## 3. Results

We enrolled sixty patients in the study: 30 were assigned to the stabilometric platform group (group 1) and 30 were assigned to the crossover group (group 2).

Baseline demographic and clinical characteristics of all patients are reported in Table 1. No variable violated the normality assumption. No statistically significant differences were observed between the two groups in any variable at the baseline.

Results from repeated measurements analysis of variance are summarized in Table 2. A significant (*p* = 0.0337) time × group interaction was found for 6MWT, indicating a difference in the effects of stabilometric platform versus crossover rehabilitation strategies. Due to this significant interaction, two separate paired *t*-tests (one for each group of patients) were carried out, demonstrating that both rehabilitation strategies improved the distance walked in 6 minutes (*p* = 0.0172 for group 1 and *p* < 0.0001 for group 2), but the improvement was better in group 2 from a statistical point of view. A different result was found for BBS, TUG, and UPDRS II, where a largely nonsignificant time × group interaction (*p* > 0.7 all) was found, revealing no differences in the effect of treatment. All these outcome variables significantly improved after the rehabilitation period (time effect *p* < 0.0001 all).

## 4. Discussion

The purpose of this study was to evaluate the effectiveness of a balance training using the crossover and to compare the results with those obtained from patients who underwent a balance treatment with a stabilometric platform.

Our results show that the crossover improves balance in Parkinsonian patients and that there are no differences in the BBS, TUG, and UPDRS improvement in comparison with the results obtained from patients who underwent a stabilometric platform treatment. Moreover, patients in the crossover group show better results in 6MWT in comparison with patients who underwent stabilometric platform training.

This is the first study that shows the efficacy of crossover on BD in Parkinsonian patients.

BD is a relevant challenge for the treatment of PD. In a precedent study, we showed the efficacy of a stabilometric platform treatment for balance disturbances in PD [5]. However, the stabilometric platforms are expensive and are not widespread devices. The possibility to find a common device as the crossover could allow the Parkinsonian patients an early, continuous, and cheap treatment that could have beneficial effects both on the development and on the control of BD. Using the crossover, the centre of mass moves continuously on three different directions. This movement determines a continuous shift of the direction of lower body force resulting in a possible stabilization and coordination benefits. The benefits of the crossover training may be also related to an implicit learning. Motor explicit and implicit learning strategies could improve rehabilitation outcomes in PD [13], and the use of feedback and cues improve the repetition of correct movements using a voluntary control which goes beyond the degenerated automatic motor mechanisms [14]. Moreover, several authors suggest that exercises in PD must incorporate specific characteristics in terms of intensity, repetition, specificity, and difficulty [9]. These characteristics are important to enhance cognitive engagement for the consolidation of learned behavior and for the changes of the dysfunctional motor circuits. Aerobic training could also restore neuroplasticity in striato-thalamic-cortical motor circuit, system involved in the automatic movements [9]. Through coordination and repetition, the crossover training combines the cognitive engagement with the aerobic training, both fundamental for motor learning mechanisms.

The biomechanics of crossover simultaneously involves legs in extending, abducting, flexing, and rotating actions improving the support base and the functionality of paravertebral muscles. This is a further reason that could explain its beneficial effect on BD. In fact, other aspects involved in BD in PD are the stooped posture, characterized by a flexion of the thoracolumbar spine due to degeneration of the paravertebral muscles [8], and a narrow base of support [6]. Both are responsible for body misalignment beyond the limit of stability, poor balance, and falls.

The repetitive and voluntary-induced movement that patients perform using crossover may determine a form of unconscious learning able to bypass the diseased basal ganglia and could act indirectly on the proprioceptive and vestibular systems. Moreover, this type of exercise counteracts the progressive course of the paravertebral muscles degeneration [15].

We have seen that patients who underwent a crossover treatment showed better results in 6MWT in comparison with those treated with stabilometric platform. The dopaminergic deficit in PD leads to an alteration in the striato-cortical pathways [16], which can affect motor unit recruitment and results in muscle weakness [17–19]. Progressive resistance exercises have been suggested as a treatment option to preserve function and health-related quality of life in PD [20, 21]. Our crossover training consists of an aerobic exercise with an increasing intensity (from low to moderate). Lima et al. have just reported an increase in muscle strength and an improvement of gait after progressive resistance training programmes in Parkinsonian patients [22]. The crossover involves the upper and lower limbs and previous studies have shown that whole body intervention programs might improve multiple specific PD impairments. In particular, Farley and Koshland [23] found an improvement in bradykinesia. It has been previously described [24] that bradykinesia/hypokinesia is the major specific PD impairment that limits the walking capacity assessed by the 6MWT [25]. We hypothesize that an improvement of bradykinesia might explain the improvement of 6MWT in crossover group.

The most important limitation of our study is the lack of a follow-up period in order to evaluate the persistence of beneficial effects and further studies are necessary to assess this issue. Moreover, it will be useful to quantify the results not only using scales but also with quantitative measurements, for example, a force plate.

In conclusion, our study shows that the crossover and the stabilometric platform have the same efficacy on BD and that crossover training might also improve walking capacity. Thus, we believe that the crossover can be proposed as a rehabilitative device for Parkinsonian patients in early and medium stage of disease because it is cheap and easily available in a common gym.

## Figures and Tables

**Table 1:** Demographic characteristics and basal clinical data in both groups.

Variable	Group 1 (stabilometric)	Group 2 (crossover)	*p* value
Age	66.6 (10.0)	65.0 (8.8)	0.500
Sex (male/female)	13/17	17/13	0.30
Height (cm)	165.7 (10.5)	166.4 (8.6)	0.76
L DOPA eq	608.7 (307.6)	740.9 (297.8)	0.059
HY	2.8 (0.4)	2.8 (0.4)	0.627
UPDRS II	15.1 (5.2)	13.9 (4.4)	0.313
BBS	45.5 (7.8)	46.3 (5.1)	0.917
TUG	12.5 (6.4)	11.0 (3.5)	0.337
6MWT	319.3 (115.1)	340.3 (87.3)	0.333

**Table 2:** Delta: value at discharge − baseline value (absolute effect size).

Variable	Group 1 Delta	Group 2 Delta	Group effect		Time effect		Time × group interaction	
			*F*(1,58)	*p*	*F*(1,58)	*p*	*F*(1,58)	*p*
BBS	7.3	7.6	0.53	0.47	170	<0.0001	0.05	0.82
UPDRS II	−5.2	−5.1	1.19	0.28	220	<0.0001	0.04	0.85
TUG	−3.3	−3.0	2.12	0.15	42	<0.0001	0.13	0.72
6MWT	68.8	103.3	2.44	0.12	118	<0.0001	4.7	0.0337
